# Insight into the bZIP gene family in *Lagenaria siceraria*: Genome and transcriptome analysis to understand gene diversification in Cucurbitaceae and the roles of *LsbZIP* gene expression and function under cold stress

**DOI:** 10.3389/fpls.2022.1128007

**Published:** 2023-02-17

**Authors:** Jian Wang, Ying Wang, Xinyi Wu, Baogen Wang, Zhongfu Lu, Liping Zhong, Guojing Li, Xiaohua Wu

**Affiliations:** ^1^ Institute of Vegetables, Zhejiang Academy of Agricultural Sciences, Hangzhou, China; ^2^ College of Horticulture Science, Zhejiang Agriculture and Forestry (A&F) University, Hangzhou, China; ^3^ State Key Laboratory for Managing Biotic and Chemical Threats to the Quality and Safety of Agro-Products, Zhejiang Academy of Agricultural Sciences, Hangzhou, China

**Keywords:** *Lagenaria siceraria*, bZIP transcription factor, Cucurbitaceae evolution, expression pattern, cold stress

## Abstract

The basic leucine zipper (bZIP) as a well-known transcription factor family, figures prominently in diverse biological and developmental processes and response to abiotic/biotic stresses. However, no knowledge of the bZIP family is available for the important edible Cucurbitaceae crop bottle gourd. Herein, we identified 65 putative *LsbZIP* genes and characterized their gene structure, phylogenetic and orthologous relationships, gene expression profiles in different tissues and cultivars, and responsive genes under cold stress. The phylogenetic tree of 16 released Cucurbitaceae plant genomes revealed the evolutionary convergence and divergence of bZIP family. Based on the specific domains, *LsbZIP* family were classified into 12 clades (A–K, S) with similar motifs and exon-intron distribution. 65 *LsbZIP* genes have undergone 19 segmental and two tandem duplication events with purifying selection. The expression profiling of *LsbZIP* genes showed tissue-specific but no cultivar-specific pattern. The cold stress-responsive candidate *LsbZIP* genes were analyzed and validated by RNA-Seq and RT-PCR, providing new insights of transcriptional regulation of bZIP family genes in bottle gourd and their potential functions in cold-tolerant variety breeding.

## Introduction

Plants are subjected to various abiotic stresses such as drought, chilling, salinity and high temperature with the increasing environmental degradation and changing weather, which extremely limits the growth and production of crops. Therefore, plants have evolved complicated stress-responsive defense tactics involving in a number of proteins, containing phosphatase, protein kinase and transcription factors (TFs), to adapt, survive and reproduce in harsh environment (Sornaraj et al., 2016). TFs, as the primary modulators in stress response, bind specific promoter regions of downstream target genes to alter transcription activity and stimulate or suppress gene expressions (Kong et al., 2015). Among more than 80 TF families, the basic leucine zipper (bZIP) family is one of the largest and diverse groups (Llorca et al., 2014; Yin et al., 2017). The bZIP domain is consist of two functional regions, the basic area for DNA binding and the leucine zipper for protein dimerization (Li et al., 2019). The basic region is highly conserved with 16 amino acid residues, including a specific motif of N-X7-R/K for nuclear localization signal and sequence-specific binding, while the leucine zipper motif is predisposed to form an alpha helical loop with dimerization specificity (Wang et al., 2011).

The bZIP TFs with plenty of diverse members have been found to be involved in the regulation of multiple biological processes, namely flower and seed development (Zhang et al., 2016; Jain et al., 2017), hormone signal transduction (Sirichandra et al., 2010), energy metabolism (Weiste et al., 2017), embryogenesis (Huang et al., 2012), photomorphogenesis (Lindemose et al., 2013), lateral root development and leaf senescence (Dankov et al., 2009). Additionally, bZIP TFs play vital roles in response to abiotic/biotic stresses including extreme temperatures, water shortage, high salinity and hormone-induced defense from pathogens (Zander et al., 2010; Lindemose et al., 2013; Sornaraj et al., 2016; Wang et al., 2020). The regulatory network of downstream genes can be controlled by the combination of bZIPs and corresponding cis-elements such as A-box (TACGTA), C-box (GACGTC), G-box (CACGTG) and abscisic acid (ABA)-responsive elements (ABRE) (CCACGTGG) (Foster et al., 1994; Zhang et al., 2008; Tang et al., 2015). In *Arabidopsis*, four bZIP factors *ABF1* (*AtbZIP35*), *ABF2* (*AtbZIP36*), *ABF3* (*AtbZIP37*) and *ABF4* (*AtbZIP38*) are predominant in regulating downstream gene expression of ABA signaling in response to osmotic stresses like drought and salt (Jakoby et al., 2002; Yoshida et al., 2015). And *TGA2* (*AtbZIP20*), *TGA5* (*AtbZIP26*) and *TGA6* (*AtbZIP45*) mediate salicylic acid (SA)-dependent pathway and meanwhile trigger ethylene and jasmonic acid (JA) pathways under abiotic stress (Zander et al., 2010). *OsbZIP1* increases pathogen resistance to *Magnaporthe grisea* in rice through SA, JA and ABA signal transduction pathway (Meng et al., 2005). *ZmbZIP17* is capable of binding ABREs and transduces stress signals through ABA pathway during germination and post-germination seedling establishment (Yang et al., 2013). Likewise, *LIP19* acts as a molecular switch for low-temperature signal transduction in rice (Shimizu et al., 2005), while *OsBZ8* positively regulates salt tolerance with ABA pathway (Mukherjee et al., 2006). With respect to number of bZIPs, 78 bZIP genes have been identified in *Arabidopsis* and classified into 13 groups (Jakoby et al., 2002; Dröge-Laser et al., 2018). So far, the genome-wide characterization of bZIP genes from many plant species have been reported, including 89 in rice (Nijhawan et al., 2008), 125 in maize (Wei et al., 2012), 247 in rapeseed (Zhou et al., 2017), 89 in barely (Pourabed et al., 2015), 92 in sorghum (Wang et al., 2011), 131 in soybean (Liao et al., 2008), 99 in poplar (Zhao et al., 2021), 56 in potato (Mirzaei et al., 2020) and 227 in wheat (Liang et al., 2022). The diversity and expansion of bZIP gene members is attributed to the whole genome, tandem and segmental duplication events (Panchy et al., 2016; Qiao et al., 2019). It’s one of the evolutionary strategies to adapt various stresses by expanding TFs family members like bZIP to achieve targeted regulation and control of complex gene expression.

Cucurbitaceae is one of the most important edible plant family in the world and encompasses nearly 1000 species from 115 genera (Schaefer et al., 2009). Bottle gourd [*Lagenaria siceraria* (Molina) Standl.], known as calabash or white-flowered gourd, is an annual Cucurbitaceae plant grown for its fruit. It is one of the world’s first cultivated plants not primarily for food, but also for use as containers, decorations, musical instruments and so on (Schlumbaum and Vandorpe, 2012). Bottle gourd is planted all over China and widely cultivated in tropical to temperate regions of the world. It is suitable to grow in conditions with moderate temperature and sufficient sunlight. However, cold stress owing to early-spring low temperature is a major abiotic stress and limiting factor for its industry (Chimonyo and Modi, 2013). Low temperature can influence many physiological and biochemical metabolisms like photosynthesis, osmotic regulation, the activity of antioxidants and so on. Breeding new bottle gourd varieties with low temperature resistance is necessary to elevate its production and quality. To accelerate the breeding process, the landscape of gene families which influence growth, development and stress response should also be comprehensively studied. As the release of our recently assembly high quality genome of bottle gourd variety “HZCG” (Xu et al., 2021), it allows us to carry out a genome-wide identification and analysis of *LsbZIP* gene family and its expression pattern under cold stress. The comparison of all bZIPs from present available Cucurbitaceae genomes in this research aims to reveal the evolution and selection in Cucurbitaceae species differentiation. This study provides the full-scale description of *LsbZIP* genes involved in response to cold stress, and lays a foundation for further molecular breeding in bottle gourd.

## Materials and methods

### Sequence retrieval and domain identification

The reference genome of bottle gourd (genome assembly ZAAS_Lsic_2.0) was obtained from our previous study (Xu et al., 2021), the query sequences 78 *AtbZIPs* (genome assembly TAIR10) from the *Arabidopsis* information resource (TAIR) (https://www.arabidopsis.org/index.jsp) were retrieved (Jakoby et al., 2002; Dröge-Laser et al., 2018), and the other genomes of selected 15 Cucurbitaceae species were downloaded from Cucurbit Genomics Database (CuGenDBv2, http://cucurbitgenomics.org/v2/) (Wang et al., 2020). A local protein database was created by TBtools v1.098769 to blast the underlying Cucurbitaceae bZIP sequences using an e value cut-off of 1e^-5^ (Chen et al., 2020). To avoid missing potential bZIPs, a hidden Markov model (HMM) file of the bZIP domain (PF00170 and PF07716) was downloaded (https://pfam.xfam.org/) and used as the template to identify bZIP sequences in the respective genome. The primary candidate sequences were further submitted in the Plant Transcription Factor Database (http://planttfdb.gao-lab.org/), SMART database (http://smart.embl.de/), NCBI CDD (https://www.ncbi.nlm.nih.gov/cdd/) for confirmation. The physicochemical properties were calculated based on the high confidence bZIPs by ProtParam (https://web.expasy.org/protparam/) (Gasteiger et al., 2003).

### Multiple alignments and phylogenetic tree construction

All *bZIP* sequences from *Arabidopsis* and 15 Cucurbitaceae were screened and aligned by Muscle algorithm (Edgar, 2004). TrimAl v1.2 was invoked to remove the unambiguous aligments using gappyout trimming mode (Capella-Gutiérrez et al., 2009). The construction of phylogenetic trees was executed with IQ-tree software, using a maximal likelihood (ML) method with 1,000 bootstrap replications (Nguyen et al., 2015). Amino acid sequences from bottle gourd, as well as 78 *AtbZIPs* were completely aligned by Clustal X2.1 and converted ALN file into MEGA 7.0.14 to build a neighbor-joining (NJ) phylogenetic tree with following parameters: poission model, and pairwise deletion for the reliability of interior branches.

### Gene distribution and duplication visualization

The chromosomal locations of *LsbZIP* genes and their tandem duplications were represented according to previous studies (Wang et al., 2022). Gene duplication events were predicted by multiple collinearity scan toolkit (MCScanX) (Wang et al., 2012). The Ka and Ks were calculated using MCScanX package to assess the selection history and divergence time of *LsbZIP* gene families. The distribution and collinearity relationships were displayed with Advanced Circos function in Tbtools.

### Prediction of conserved motifs and exon/intron structure

To better decipher the structure diversity of *LsbZIP* genes, motifs were detected using the Multiple Expectation Maximization for Motif Elicitation (MEME)/Motif Alignment and Search Tool (MAST) system (http://meme-suite.org/) with default set (Bailey et al., 2009). The exon-intron structure and the intron phases of *bZIP* gene family were visualized by the online program Gene Structure Display Server 2.0(GSDS, http://gsds.gao-lab.org/) (Hu et al., 2015).

### Orthologous gene clusters analysis of eight selected species

Eight common and important species genomes namely *Arabidopsis thaliana*, *Brassica napus*, *Cucumis sativus*, *Glycine max*, *Oryza sativa*, *Solanum lycopersicum*, *Vitis vinifera* and *Lagenaria siceraria* were blasted through Ortho Venn 2.0 web server (https://orthovenn2.bioinfotoolkits.net/home). 65 *LsbZIP* protein sequences were uploaded and analyzed with e-value 1e-5 and inflation value of 1.5 (Wang et al., 2022).

### *Ab initio* prediction of the core promoter in *LsbZIPs*

The putative promoter sequences were extracted from 2000 base pair (bp) upstream of the transcriptional starting site (ATG) of genomic DNA sequences. The cis-regulatory elements (CREs) were obtained by introducing promoter sequences into the PlantCARE database (http://bioinformatics.psb.ugent.be/webtools/plantcare/html/).

### The expression patterns analysis of *LsbZIP* genes in different tissues and varieties

To determine the gene expression profiles of bZIP family, RNA-Seq data of various tissues including leaf, root, stem, flower and fruit were downloaded from NCBI sequence read archive (SRA) with accession SRP107894 (Wu et al., 2017). A collection of re-sequencing data of 5 bottle gourd accessions were also downloaded from SRP095913 (Xu et al., 2021). The expression of each *LsbZIP* gene was quantified with transcripts per million (TPM). The transcript abundances were output by kallisto in TPM units. Normalization (log_2_) and heat-maps were generated using Tbtools software.

### Plant materials and treatments

‘YZ’, a bottle gourd variety of rootstock type tolerance to low temperature, was used in this experiment. Healthy and sterilized seeds were germinated and sown in plastic pots. The seedings were placed in growth chamber at 28°C/22°C (16 h/8 h) day/night temperatures with a relative humidity of 75%. At 2~3 leaf stage, uniform seedlings were divided into two groups for room temperature (24°C) or cold treatment (4°C). The leaf samples were harvested for gene expression validation at 0, 1, 6, 12, 24 h after cold treatment. And three biological replications were used in this study.

### Transcriptome and qRT-PCR analysis

The transcriptional profile of bZIP genes of bottle gourd under cold stress were downloaded under accession PRJNA553072 from our previous study (Wang et al., 2020). The differentially expressed genes (DEGs) of *LsbZIPs* were quantified with fragments per kilobase of transcript per million mapped fragments (FPKM) method between control and cold treatment. DEGs were screened according the criteria (false discovery rate <0.5 and absolute fold change ≥2). For cold-induced *LsbZIPs* expression, total RNA was extracted from RNAprep Pure Plant Kit (Tiangen, China). *TuB-α* gene (BG_GLEAN_10019523) was used as the internal control gene in bottle gourd (Li et al., 2021). qRT-PCR was performed on a Bio-Rad CFX96 (BIO RAD, USA) with SuperReal PreMix Plus/SYBR Green (Tiangen, China). Triplicate replications were performed for analysis. The gene relative expression level was calculated by the 2^–ΔΔCt^ method. All primers used for qRT-PCR are listed in **Supplementary Table S1**.

## Results and discussions

### Systematic identification and classification of *bZIPs*

**Figure 1:**
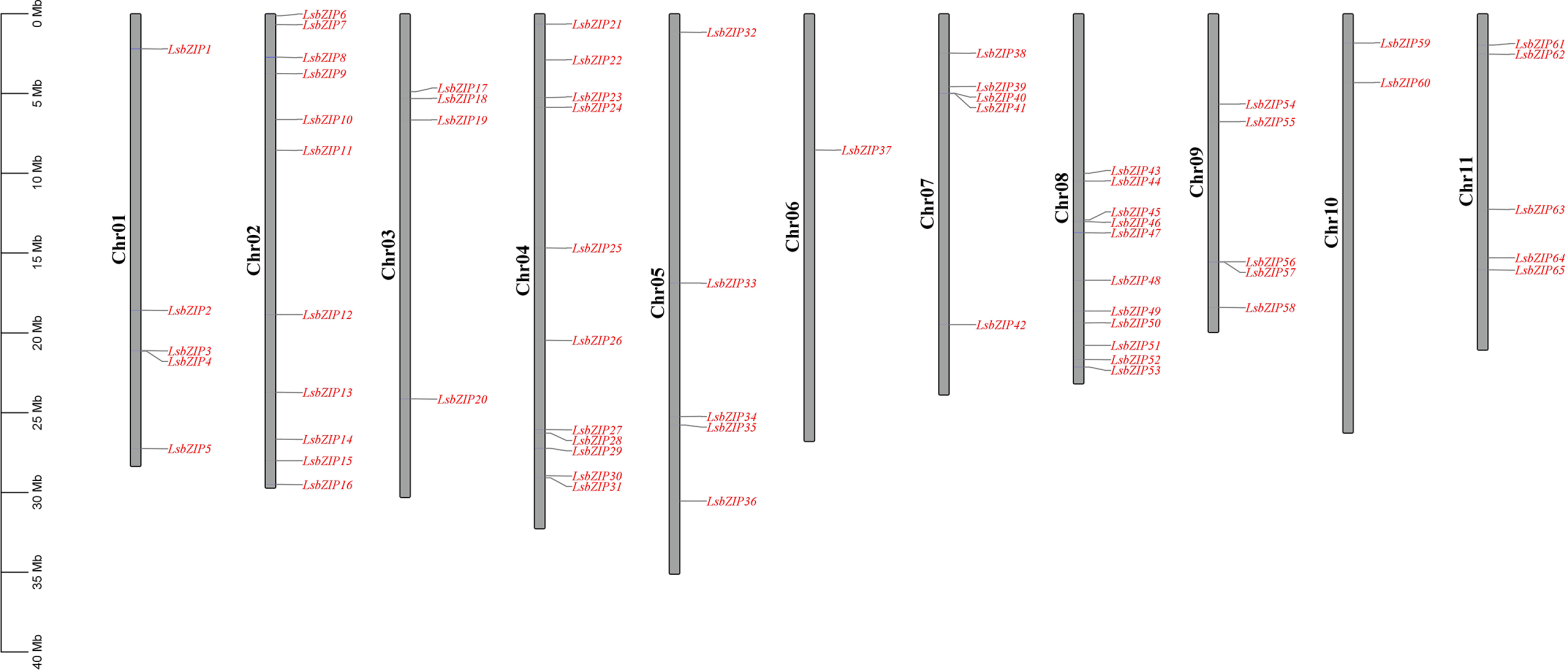
The distribution of bZIP genes on the bottle gourd chromosomes.

So far two genomes of bottle gourd inbred lines, HZCG (food-type, China) assembled with Illumina paired-end and USVL1VR-Ls (rootstock-type, India) assembled with PacBio and BioNano, have been released (Wu et al., 2017; Xu et al., 2021). Based on the specificity of bZIP family domain, 65 and 60 bZIP genes were identified in HZCG and USVL1VR-Ls, respectively (**Table S2**, **Figure S1**). The comparison of bZIP genes in two bottle gourd reference genomes showed that each bZIP in USVL1VR-Ls was able to find its homolog in HZCG (**Supplementary Figure S1**). Thus, we selected 65 bZIPs in HZCG genome for our subsequent analysis and named them as *LsbZIP1*~*LsbZIP65* according to their location (**Table S2**). Generally, 65 *LsbZIPs* were roughly spread equally across 11 all chromosomes (Chr) while only Chr 6 contained one bZIP gene and Chr 10 contained two bZIP genes (**Figure 1**). The physicochemical parameters showed that the length of amino acid (AA) residues ranged from 129 AA (*LsbZIP36*) to 1225 AA (*LsbZIP8*), with isoelectric point (pI) ranging from 4.55 (*LsbZIP6*) to 9.89 (*LsbZIP33*) (**Supplementary Table S2**). The molecular weight (MW) ranged from 15.14 kDa (*LsbZIP36*) to 135.15 kDa (*LsbZIP8*) with average 39.74 kDa. In addition, the prediction of subcellular localization of all *LsbZIP* proteins were distributed in nucleus except *LsbZIP8* locating on plasma membrane and *LsbZIP34* locating in cytoplasmic zone.

**Figure 2:**
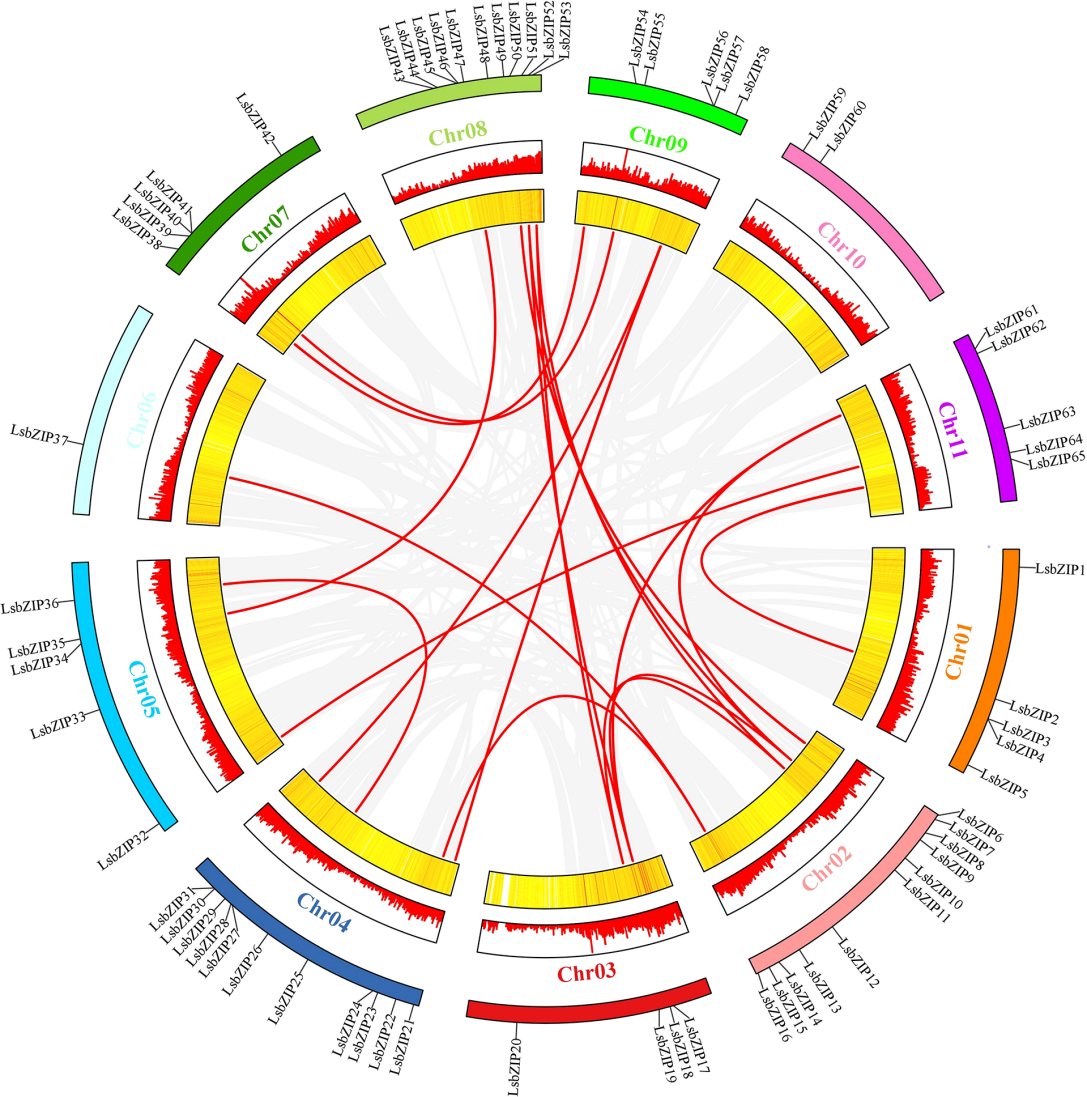
Interchromosomal synteny of linked *LsbZIP* genes. The red bars and yellow color strips indicated the gene density in the chromosomes.

For the gene duplication events, 19 gene pairs were segmentally duplicated on all chromosomes except Chr 10 (**Figure 2**; **Table 1**). There were only two tandem duplication events with gene pairs (*LsbZIP40*/*LsbZIP41*, *HG_GLEAN_10002003*/*LsbZIP62*) in bottle gourd. To estimate the selective pattern, Ka/Ks is calculated to uncover the diversity of duplicated gene pairs in the evolution. In this study, all duplicated gene pairs were subjected to strong purifying selection for the purpose of eliminating detrimental mutations as Ka/Ks<1 while 8 duplicated gene pairs showed NaN caused by high sequence divergence value (**Table 1**).

**Table 1:** Ka/Ks calculation for the duplication of bZIP gene pairs in bottle gourd.

Duplicated gene 1	Duplicated gene 2	Ka	Ks	Ka/Ks	Duplication type
*LsbZIP40*	*LsbZIP41*	0.095567774	0.46292708	0.206442393	Tandem
*HG_GLEAN_10002003*	*LsbZIP62*	0.20465024	0.746788399	0.274040465	Tandem
*LsbZIP2*	*LsbZIP65*	0.173528729	1.118537237	0.155138983	Segmental
*LsbZIP8*	*LsbZIP50*	0.205161542	3.729959631	0.05500369	Segmental
*LsbZIP10*	*LsbZIP17*	0.398267583	NaN	NaN	Segmental
*LsbZIP10*	*LsbZIP51*	0.309843954	NaN	NaN	Segmental
*LsbZIP10*	*LsbZIP62*	0.384704842	2.136583395	0.180056085	Segmental
*LsbZIP11*	*LsbZIP19*	0.669456185	NaN	NaN	Segmental
*LsbZIP11*	*LsbZIP53*	0.457676449	2.188328049	0.209144351	Segmental
*LsbZIP14*	*LsbZIP22*	0.342001797	NaN	NaN	Segmental
*LsbZIP14*	*LsbZIP37*	0.304349709	1.27762649	0.238214933	Segmental
*LsbZIP17*	*LsbZIP51*	0.412887924	1.819003474	0.226985781	Segmental
*LsbZIP17*	*LsbZIP62*	0.37037958	1.513609512	0.244699559	Segmental
*LsbZIP19*	*LsbZIP53*	0.664505207	NaN	NaN	Segmental
*LsbZIP21*	*LsbZIP57*	0.290708373	1.870059385	0.155454086	Segmental
*LsbZIP26*	*LsbZIP36*	0.264737608	NaN	NaN	Segmental
*LsbZIP30*	*LsbZIP56*	0.547629151	NaN	NaN	Segmental
*LsbZIP32*	*LsbZIP63*	0.696047991	NaN	NaN	Segmental
*LsbZIP34*	*LsbZIP46*	0.307254005	1.778039766	0.172804912	Segmental
*LsbZIP38*	*HG_GLEAN_10000143*	0.423630263	3.164151086	0.133884335	Segmental
*LsbZIP39*	*LsbZIP55*	0.135245327	1.184936605	0.114137184	Segmental

NaN means high sequence divergence value (pS ≥ 0.75).

### Phylogenetic analysis of Cucurbitaceae species

**Figure 3:**
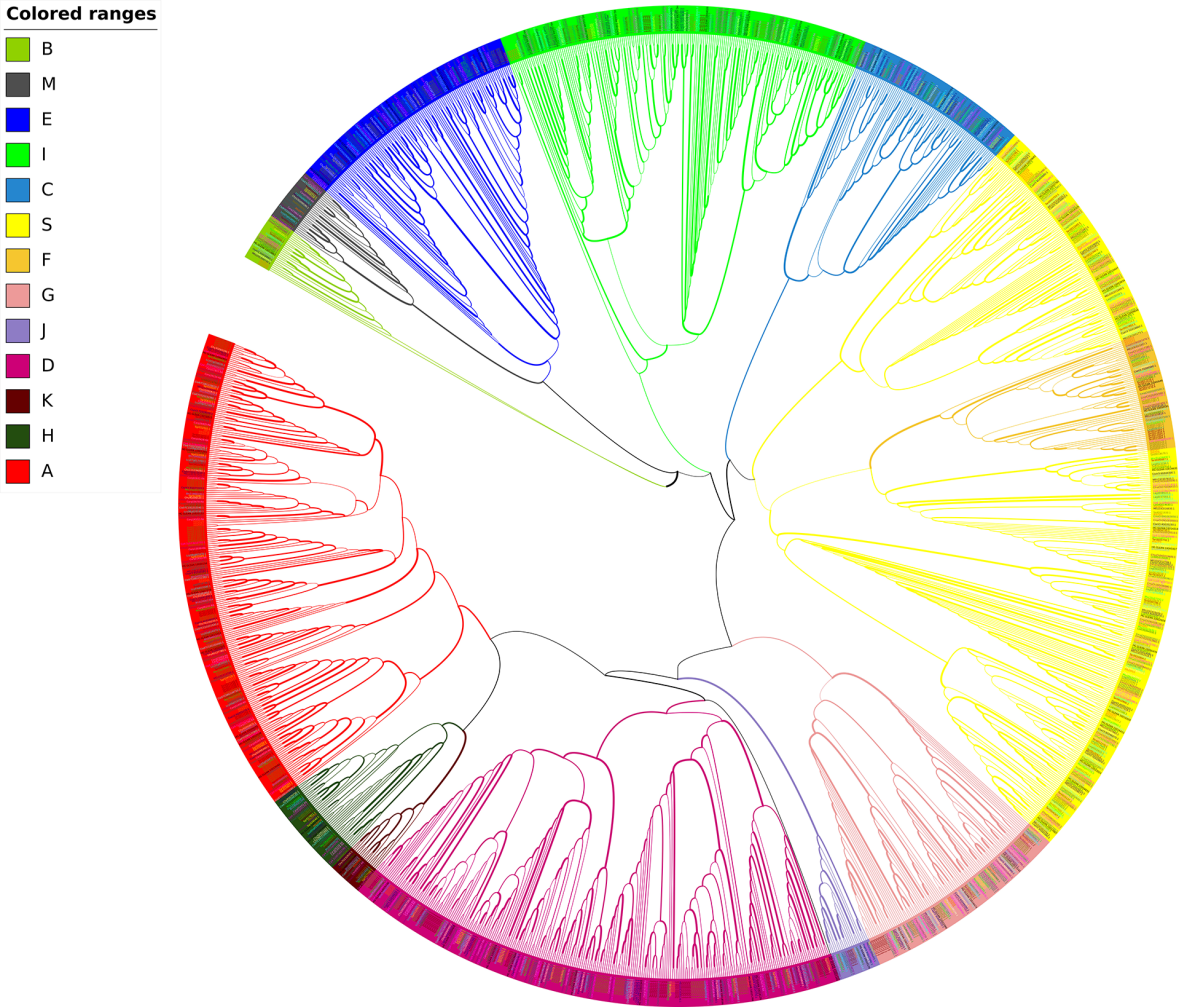
Unrooted Maximum likelihood phylogenetic tree of bZIP genes in 16 Cucurbitaceae species. Different colors represent bZIPs in different clades according to Dröge-Laser et al. (2018).

Based on the specific domains bZIP_1 (PF00170) and bZIP_2 (PF07716), we finally identified and confirmed 1510 sequences from 17 Cucurbitaceae species including two different bottle gourd genomes (**Tables 2**, **S3**, **S4**; **Figure 3**). Apart from the splicing variants found in *Sechium edule* and *Trichosanthes anguina*, only 1235 unique *bZIP* sequences were retrieved and grouped into 13 subfamilies in Cucurbit genomes. Generally, clade A, D, E, I and S occupied the numerous bZIP members exceeding 100 while other clades B, J, K contained limited numbers of bZIP homologs less than 20 (**Table 2**). 11 of 13 clades were existed in all selected cucurbit crops except clades B, K and M. Clade B and K usually owned 1~3 and only one bZIP gene in 15 species (not found in cushaw pumpkin and ridge gourd) respectively, and clade M was consist of 1~ 3 bZIP members in 12 species (not identified in wax, cucumber, bottle gourd and bitter gourd). The number showed diverse distribution not only in different clades, but also in different species. The total number of bZIPs in 16 species could be divided into two categories. One type mostly belonged to Cucurbita genera including *C. argyosperma*, *C. maxima*, *C. moschata* and *C. pepo* besides *S. edule*. The gene family number in this class was approximately two-fold than it in another class reaching the amount of average 65 members (**Table 2**). The majority of all clades were quite distinct from each other, but clade F was sandwiched between two separated clades S.

**Table 2:** bZIP family members of 16 Cucurbitaceae plant species and their phylogenetic classifications.

Plant species	Common name	Number of *bZIP* genes	Group classfication													Reference
			A	B	C	D	E	F	G	H	I	J	K	M	S	
*Benincasa hispida*	Wax gourd	62	9	1	4	9	5	2	4	2	8	1	1	–	16	In this study
*Citrullus lanatus*	Watermelon	66	11^c^	1	4	9^c^	5	2	4	2	8	1	1	2^c^	16^c^	Yang et al. (2019)
*Cucumis hystrix*	Wild cucumber	62	9	1	4	8	7	1	5	3	7	1	1	1	14	In this study
*Cucumis melo*	Melon	62	9	1	4	9	5	2	3	2	8	1	1	1	16	In this study
*Cucumis sativus*	Cucumber	65^c^	10	1	4	10	6	2	4	2	9	1	1	–	15	Baloglu et al. (2014)
*Cucurbita argyrosperma*	Cushaw pumpkin	101	17	–	7	14	9	4	7	3	9	1	1	3	26	In this study
*Cucurbita maxima*	Winter squash	109	16	2	6	17	10	4	8	3	15	1	1	3	23	In this study
*Cucurbita moschata*	Pumpkin	108	17	2	6	16	8	4	7	4	14	2	1	3	24	In this study
*Cucurbita pepo*	Zucchini	93	8	3	7	14	8	4	8	3	11	1	1	2	23	In this study
*Lagenaria siceraria*	Bottle gourd	65	10	1	3	9	5	3	4	2	9	1	1	–	17	In this study
*Luffa acutangula*	Ridge gourd	68	10	1	4	10	6	2	4	2	9	1	–	2	17	In this study
*Luffa cylindrica*	Sponge gourd	68	11	1	4	9	6	2	4	2	8	1	1	2	17	In this study
*Momordica charantia*	Bitter gourd	63	9	1	4	9	7	2	6	2	8	1	1	–	13	In this study
*Sechium edule*	Chayote	102^a^	15	2	7	14	9	4	8	2	15	1	1	2	22	In this study
*Siraitia grosvenorii*	Monkfruit	75	11	1	6	12	4	1	5	2	9	1	1	2	20	In this study
*Trichosanthes anguina*	Snake gourd	66^a^	11^b^	1	4	9	6	2	3	2	8	1^b^	1	2	16	In this study
Total		1235	183	20	78	178	106	41	84	38	155	17	15	25	295	

^a^: the occurrence of additional splicing variants; ^b^: splicing variants belong to different clades of bZIPs; ^c^: values differ from previously published numbers.

### Gene structure and motif analysis

**Figure 4:**
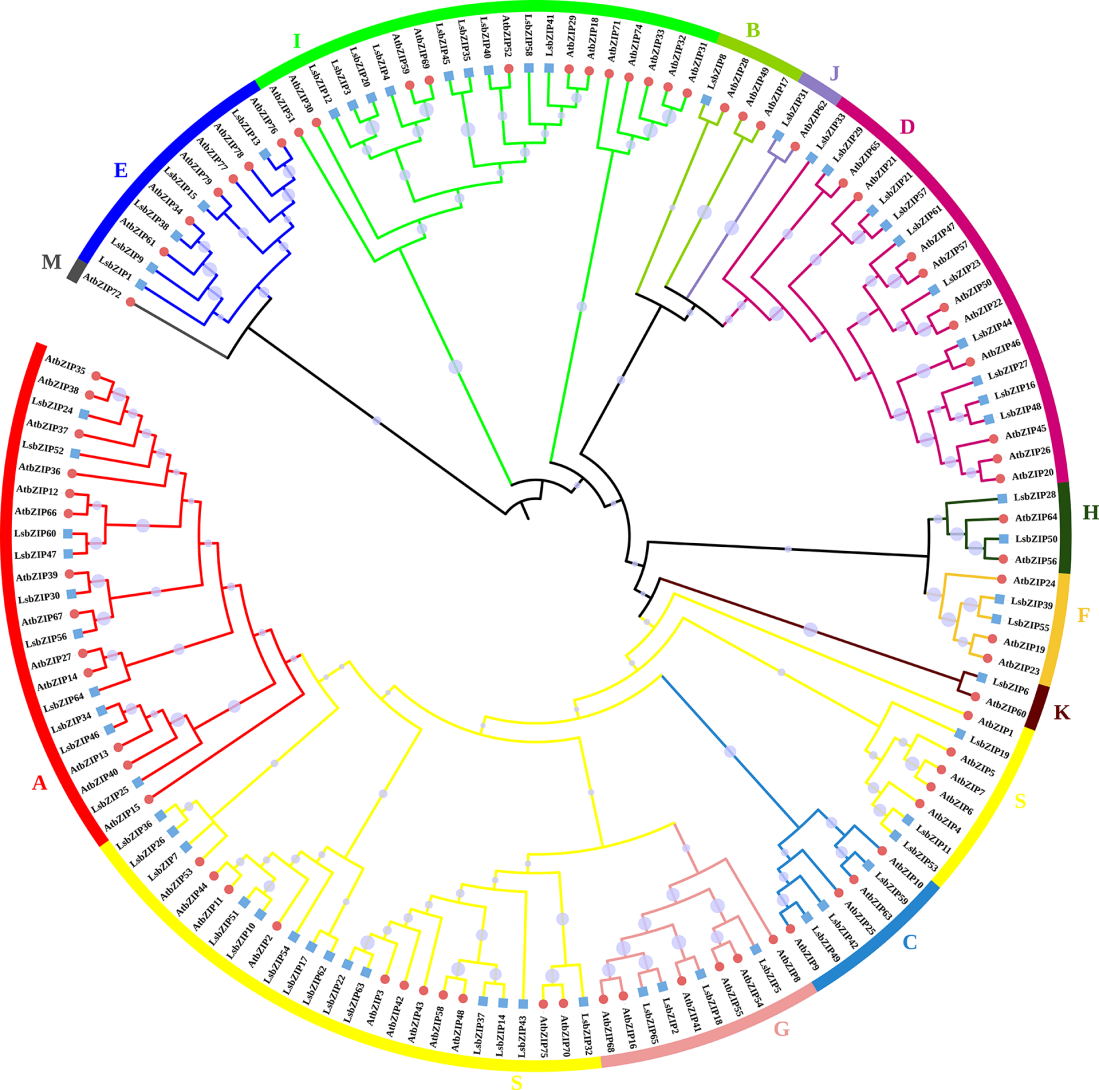
Phylogenetic tree of bZIP genes from *Arabidopsis* and bottle gourd. The tree is constructed by Neighbor-Joining method with bootstrap values of 1000 replicates. Different colored branches and stripes indicate different subgroups. The red circles and blue squares represent bZIP genes from *Arabidopsis* and bottle gourd, respectively.

According to the classification criterion of *AtbZIPs* by Dröge-Laser et al. (2018), 65 *LsbZIPs* were divided into 12 clades (LsbZIP-A, LsbZIP-B, LsbZIP-C, LsbZIP-D, LsbZIP-E, LsbZIP-F, LsbZIP-G, LsbZIP-H, LsbZIP-I, LsbZIP-J, LsbZIP-K and LsbZIP-S) based on their characterized domains (**Figure 4**). To recapitulate briefly, different clades had diverse phylogenetic clustering. For the relatively small clade like clade J, H and K, bZIPs from two species showed highly homologous with nearly 100 bootstrap value. In some clades like clade B, I and S, the whole clade can be further separated into two or three subfamilies because of the dissimilar divergences. On the other hand, *LsbZIPs* didn’t present one to one mapping relationship with *AtbZIPs*, indicating the selective evolution from gene duplication.

**Figure 5:**
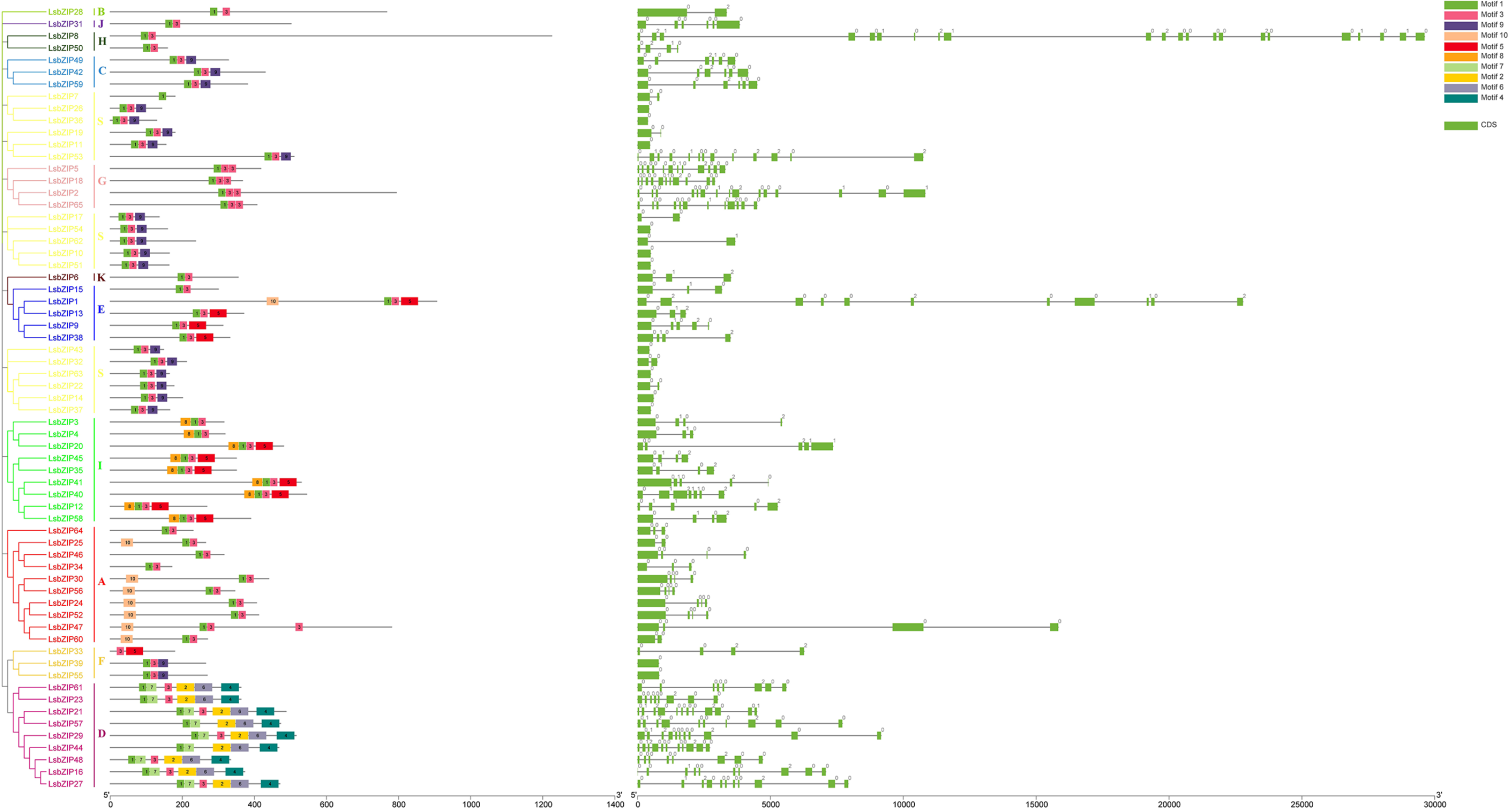
Conserved motifs and gene structures of *LsbZIP* genes in the bottle gourd. Left: 10 Motifs are indicated by different colored boxes. Right: Green boxes indicate the exons, and grey lines indicate the introns. Different numbers indicate the phase of introns.

To better depict the specific domain, the phylogeny, exon/intron structure and conserved motifs distribution were integrated in **Figure 5**. The phylogenetic classification was consistent with previous results. According to MEME analysis, 10 highly conserved motifs were identified in *LsbZIPs*. And all 65 *bZIPs* roughly split into 7 types in consideration of the number and order of motifs. Four clades (B, J, H and K) shared the same distribution pattern with motif 1 and motif 3. *LsbZIPs* containing motif 9 at the C-terminal end were found in type C, S, F while clade A had motif 10 at the N-terminal end. Motifs 5 and 8 can be detected only in clade I, and then 4 motifs (7, 2, 6, 4) can be identified only in clade D. In addition, Clade G owned a replication with motif 3. The results indicated that members classified into the same clade mostly shared the same or similar conserved motifs (**Figure 5** and **Table S5**). The distributed positions of exons/introns on *LsbZIP* genes were investigated by GSDS, to gain further insight into the structure diversity in bottle gourd. The number of exons ranged from 1 to 22, and clade G and D had relative more exons than other clades. The phylogenetic classification of *LsbZIPs* was not obviously consistent with exon/intron distribution. However, some genes in the same type shared similar structures, such clade C possessed six exons and five introns.

### Orthologous clusters comparisons in different species

**Figure 6:**
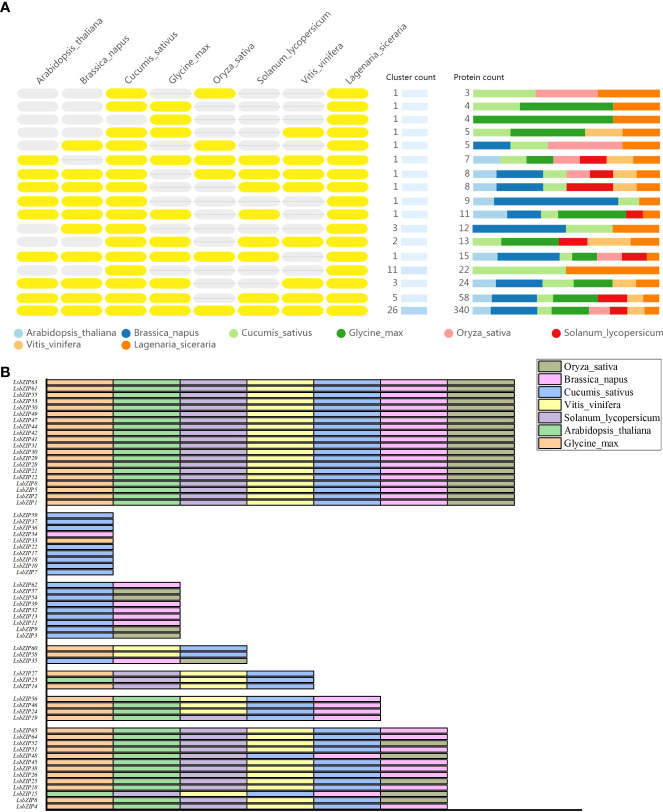
Comparative analysis of orthologous gene clusters among multiple species. **(A)** Yellow ovals on the left side represent the presence of orthologous genes while grey ovals represent the absence of orthologous genes. Different colors bars on the right side indicate the number and ratio of orthologous proteins in 8 plant species. **(B)** Layout of the different orthologous gene pairs. Each species was marked with different color.

The comparison and annotation of orthologous gene clusters among multiple species were displayed in **Figure 6A** (https://orthovenn2.bioinfotoolkits.net/task/result/3515a11fe06d38df8aca0e62cc1739d9). The number of orthologous proteins in each cluster varied from 4 to 352. In summary, a total of 60 orthologous proteins in *C. sativus* were predicted to have similar conserved domain with *LsbZIPs*. *O. sativa* had the lowest homology with 30 proteins while the orthologous proteins in the rest species varied from 37 (*S. lycopersicum*) to 42 (*G. max*). 8 *LsbZIPs* were only orthologous in *C. sativus*, *LsbZIP33* and *LsbZIP34* was only orthologous in *G. max* and *B. napus*, respectively (**Figure 6B**, **Table S6**). 20 *LsbZIP* proteins were able to find their orthologous proteins in each species. 26 clusters including 352 proteins were overlapped in all 8 species and 26 proteins belonged to *L. siceraria* were corresponding to orthologous proteins, respectively (**Figure 6A**). 3 singleton proteins (*LsbZIP43*, *LsbZIP20*, *LsbZIP40*) were identified to have no orthologs in any of these species.

### Cis-regulatory elements analysis of *LsbZIP* genes

**Figure 7:**
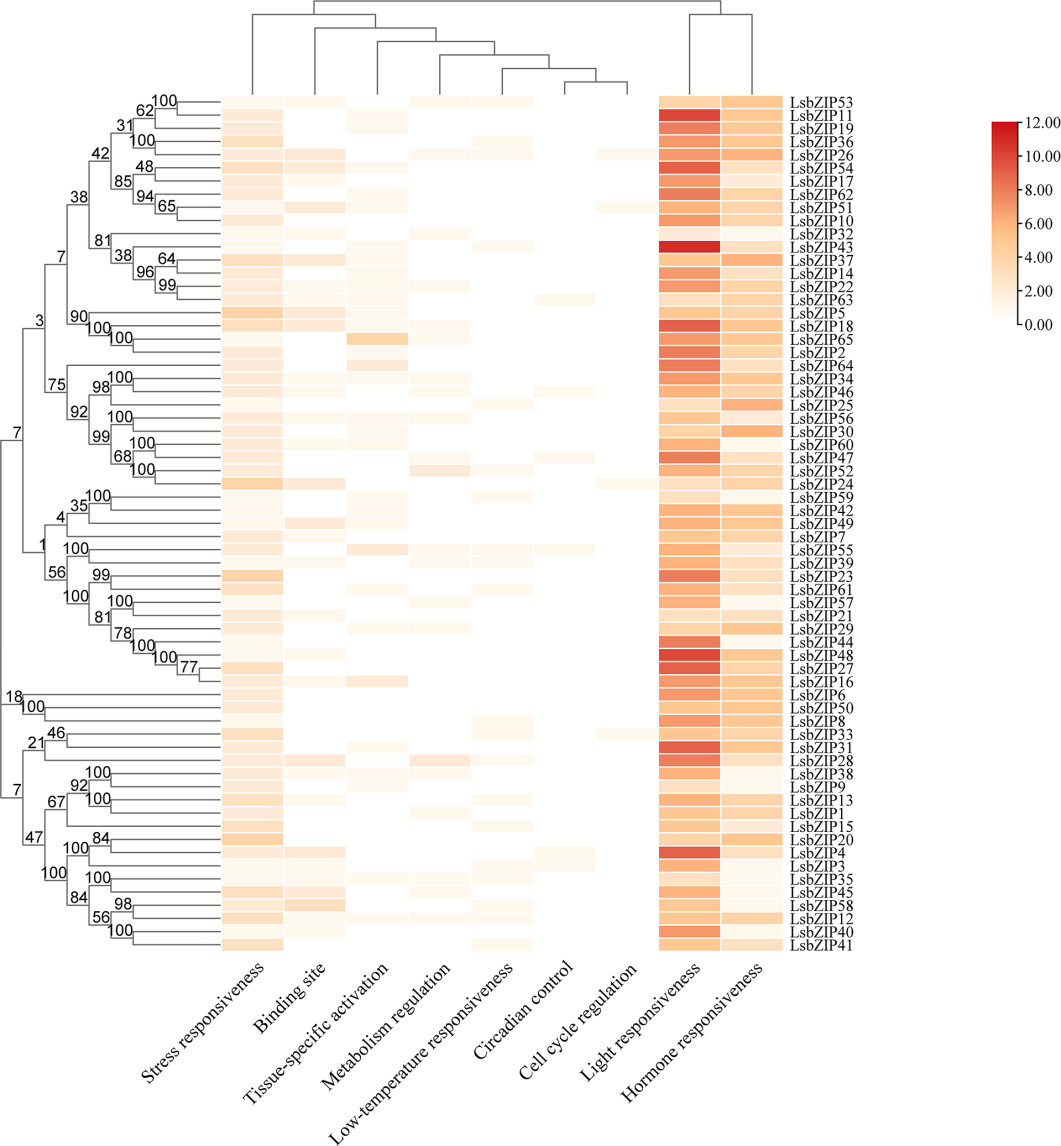
Cis-acting regulatory elements in the promoters of *LsbZIPs*.

To further understand the transcriptional regulation of bZIP genes in bottle gourd, 2000 bp sequences upstream of the translation initiation site (ATG) were extracted to predict cis-elements by PlantCARE database. In a summary, we identified 64 cis-acting elements which can be grouped into 9 different responsive processes based on the diverse functions (**Table S7**, **Figure 7**). Besides the core promoter elements (CAAT-box and TATA-box) found in all 65 *bZIP* genes, the column clustering had a certain degree of preferences. CREs involved in light responsiveness, hormone responsiveness and stress responsive were ubiquitous in each *LsbZIP*, accounting for the majority of putative promoters. Three genes (*LsbZIP11*, *LsbZIP43*, *LsbZIP48*) had more than 10 different light-responsive CREs. The numbers of concerned CREs in stress responsiveness contained low-temperature responsive elements (LTR) in 20 *LsbZIPs*, anaerobic-responsive element (ARE) in 57 *LsbZIPs*, wound-responsive element (WUN-motif) in 7 *LsbZIPs*. Twelve hormone responsive elements were found, in which included abscisic acid responsiveness element (ABRE) in 41 *LsbZIPs*, auxin-responsive element (AuxRR-core, TGA-element) in 6 *LsbZIPs*, gibberellin-responsive element (GARE-motif, P-box, and TATC-box) in 31 *LsbZIPs*, salicylic acid responsiveness (SARE, TCA-element) and MeJA-responsiveness (CGTCA-motif and TGACG-motif) in 35 and 41 *LsbZIPs*, respectively (**Table S7**). Moreover, circadian control was found in 6 *LsbZIPs* as well as zein metabolism regulation (O2-site) and flavonoid biosynthetic gene regulation (MBSI) were found in 15 and 4 *LsbZIPs*, respectively. The results demonstrated that bZIP TFs acted as an important role not only in the regulation of multiple biological processes, but also in response to many abiotic stresses.

### Expression patterns of *LsbZIPs* in various tissues and cultivars

**Figure 8:**
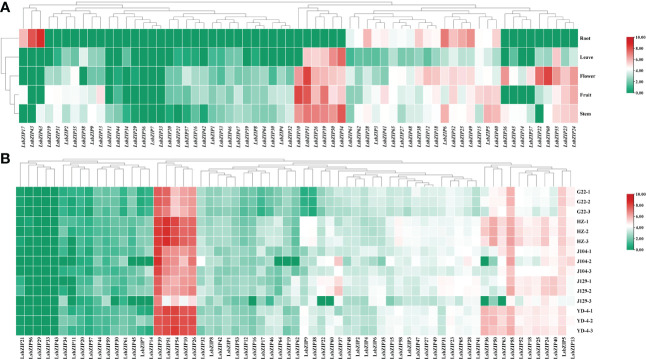
Heatmaps of *LsbZIP* genes displaying tissue-specific and cultivar-specific expression. **(A)** Hierarchical clustering of expression profile of bZIP genes in different tissues (root, leaf, flower, fruit, stem) of bottle gourd under normal condition. **(B)** The expression profile of bZIP genes in 5 bottle gourd varieties (G22, HZ, J104, J129, YD-4) under normal condition. Data were transformed with a log_2_(TPM+1) transformation.

To dissect the tissue-specific expression of *LsbZIP* genes, the expression of 65 *LsbZIPs* were determined in five tissues (root, leaf, flower, fruit and stem) from RNA-Seq data SRP107894 (Wu et al., 2017). As shown in **Figure 8A**, four genes (*LsbZIP29, LsbZIP56, LsbZIP7, LsbZIP33*) were found not totally expressed in all tissues under normal conditions, indicating that they may be pseudogenes or act in response to abiotic/biotic stresses. And 17 genes were expressed at a low level (log_2_(TPM+1)<3), especially in roots and leaves. Meanwhile, 6 genes (*LsbZIP10, LsbZIP51, LsbZIP26, LsbZIP39, LsbZIP50, LsbZIP54*) were highly expressed in leaves, flowers, fruits and stems except in roots. These genes may be involved in the development process of various organs. In roots, 44 of 65 identified *LsbZIP* genes were regarded as silent while 11 *LsbZIP* genes exhibited high transcript expression level (**Table S8**). Three genes (*LsbZIP17, LsbZIP43, LsbZIP63*) were root-specific expressed while four genes (*LsbZIP36, LsbZIP45, LsbZIP14 and LsbZIP37*) were relatively high induced in flowers and stems. To sum it up, tissue-specific genes with high expressions might be involved in corresponding tissue defense system. And the illustrated heatmap showed that *LsbZIPs* were most active in flower and not relatively active in leaves. To further discuss the expression patterns in different varieties, our previous RNA-Seq data with five bottle gourd accession were used in this study (Xu et al., 2021). The results showed that 5 genes (*LsbZIP21*, *LsbZIP56, LsbZIP29, LsbZIP33, LsbZIP43*) were rarely expressed in all cultivars, similar to the tendency in **Figure 8B**. The genes with high expression level in different cultivars were exactly same in different tissues, demonstrating these genes played important roles in manipulating normal growth and development in plants. However, three genes (*LsbZIP36, LsbZIP50, LsbZIP63*) were highly expressed in variety HZ and YD-4 whilst low expression in G22, J104 and J129. And *LsbZIP37* had relative high transcript abundance in J104 and J129 than in other 3 cultivars (**Table S9**). It revealed that a few *LsbZIP* genes had cultivar-specific expression, to a certain extent.

### Expression of *LsbZIP* genes under cold stress

**Figure 9:**
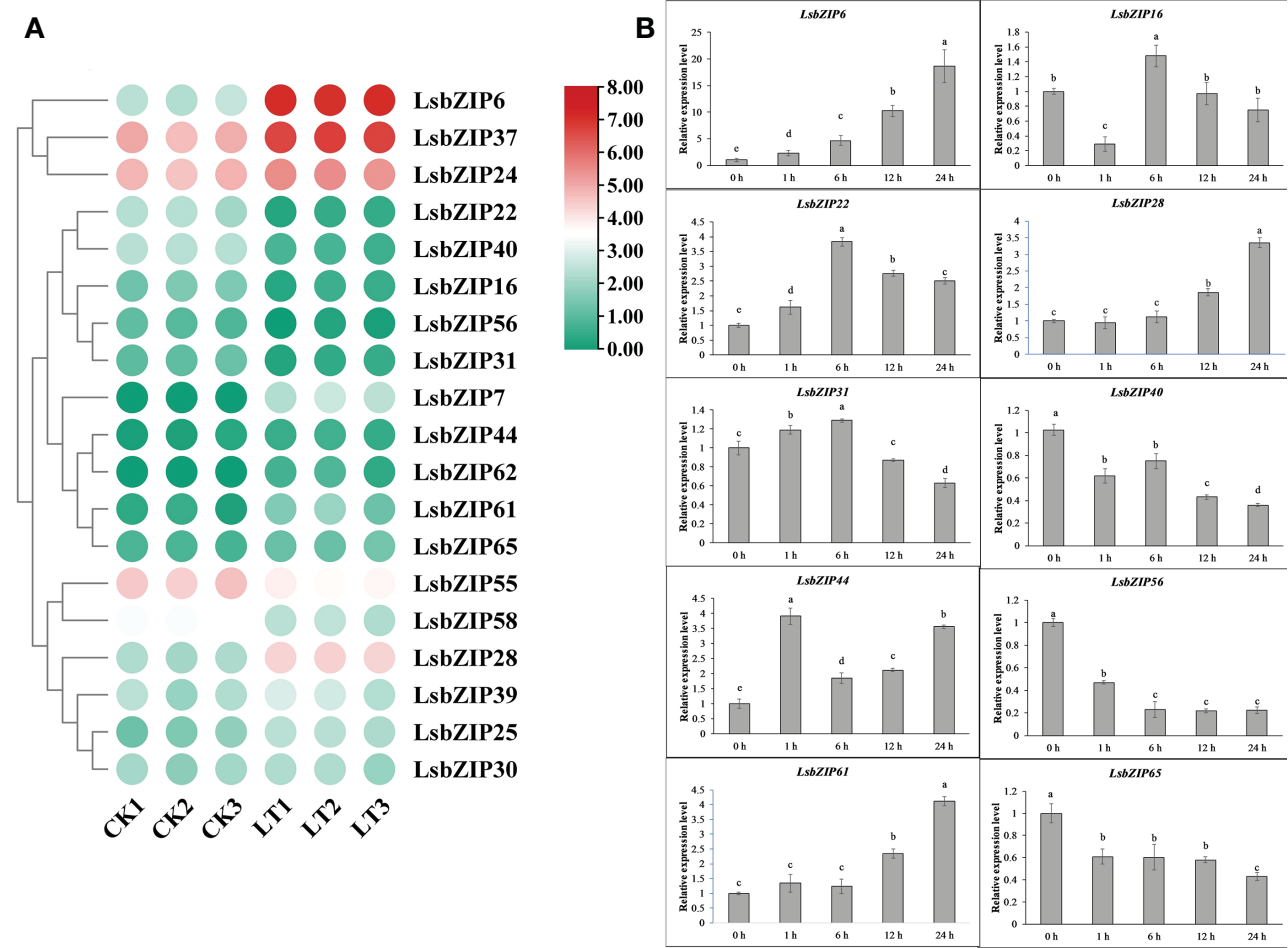
Expression profile of *LsbZIP* genes in leaves under cold stress. **(A)** The expression levels of DEGs in leaves under cold stress. **(B)** Relative expression of selected 10 *LsbZIP* genes in response to cold stress at 5 time points. Mean ± SE of three replicates represent the significant difference with LSD test. The data followed by the different letter in the graph are significant at P≥0.05.

To investigate early changes in gene expression, we retrieved the transcriptional data under the accession number PRJNA553072 in the bottle gourd cultivar “YZ”, which is a rootstock type showing high tolerance to low temperature (Wang et al., 2020). The differentially expressed genes (DEGs) in accordance with false discovery rate <0.5 and absolute fold change ≥2 were selected and shown in **Figure 9A**. In general, 19 *LsbZIPs* were detected in YZ under low temperature treatment for 2 days. 7 genes were significantly down-regulated and 10 genes were up-regulated remarkably (**Figure 9A** and **Table S10**). Intriguingly, two genes (*LsbZIP7* and *LsbZIP62*) were not expressed at room temperature similar to the results found in **Figure 8A**. And *LsbZIP7* was highly induced after low temperature treatment while *LsbZIP62* was slightly induced, as well. Three genes *LsbZIP55*, *LsbZIP37*, and *LsbZIP24* which kept relative high gene expression exhibited opposite expression patterns. The expression of *LsbZIP55* cut in half, but the expression level of *LsbZIP37* and *LsbZIP24* increased 2-3 folds compared to respective control (**Table S10**). In addition, another three genes namely *LsbZIP28*, *LsbZIP61*, *LsbZIP6* increased their expression up to 5, 7, 30 times respectively in response to cold stress. Five genes (*LsbZIP56*, *LsbZIP40*, *LsbZIP16*, *LsbZIP31* and *LsbZIP22*) were dramatically down-regulated by low temperature. To gain insight into the changing trends in response to cold stress, 10 selected *LsbZIP* genes were determined by qRT-PCR to validate the RNA-Seq data (**Figure 9B**). The leaves were harvested at 5 different time-points (0h, 1h, 6h, 12h, 24h) after cold treatment at 4°C. The plants without 0 h cold treatment were considered as control. The relative expression level of selected *LsbZIP* genes was evaluated by the 2^–ΔΔCt^ method. Statistical significance was detected by LSD test using SPSS v16.0 (SPSS, Inc., Chicago, IL, USA). 8 genes were activated fast within one hour cold stress, indicating the responsive rate varied in different gene expression. *LsbZIP6* was quickly induced to be remarkably expressed at 1 hour after treatment (HAT) and continuously rise up to 20 folds than 0 HAT. The parallel increasing pattern was observed in *LsbZIP61* and *LsbZIP28*, but the expression of these two genes were initiated to ascend 12 HAT. Three genes (*LsbZIP16*, *LsbZIP22* and *LsbZIP31*) presented earlier increase and later decrease trend. On the other hand, three genes including *LsbZIP40*, *LsbZIP56*, *LsbZIP65* were down-regulated within 1 HAT and subsequently inhibited all the time till 24 HAT. In addition, only one gene *LsbZIP44* showed a distinct expression pattern. It was highly expressed within 1 HAT, then suppressed after 6 h cold treatment. Interestingly, *LsbZIP44* was again induced, followed by gradual increase at 12 and 24 h.

## Discussion

Increasing evidences have suggested that bZIP TFs play vital roles in many biological and physiological processes and regulating signal transductions in response to various abiotic and biotic stresses, such as seed germination (Toh et al., 2012), flower development (Strathmann et al., 2001), cold tolerance (Ritonga and Chen, 2020), salt stress (Wang et al., 2010) and pathogen defense (Thurow et al., 2005), etc. So far, a number of plant species have systematically analyzed bZIP family genes in the genome-wide scale, including some crucial crops like *A. thaliana* (78) (Dröge-Laser et al., 2018), *O. sativa* (89) (Nijhawan et al., 2008), *Z. mays* (125) (Wei et al., 2012) and a few cucurbit vegetables cucumber (64) (Baloglu et al., 2014) and watermelon (62) (Yang et al., 2019). However, the genome-wide characterization of bZIP family genes in *Lagenaria siceraria* has not been reported. In this study, we compared the former published bottle gourd genome to our newly assembled genome and finally identified 65 unique *LsbZIP* sequences.

Cucurbitaceous plants, also known as the gourd family, are one of the most important edible plant families and have great economic value. Currently, at least 16 genomes of Cucurbitaceae species including diverse varieties in each species have been released (Wang et al., 2020). To dig deeply into the evolution and divergence in Cucurbit, the available known cucurbit genomes were collected and exhaustively searched for the putative bZIP genes. A total number of 1235 non-redundant protein sequences from 16 Cucurbitaceae species were subdivided into 13 clades with well-supported bootstrap values (**Figure 3**, **Table S4**). In clade B, three *AtbZIPs* (*AtbZIP17*, *AtbZIP49*, *AtbZIP28*) formed a small unique cluster independent from other Cucurbitaceae bZIPs. This scenario also emerged in clade A, F, I, suggesting that these group-specific sequence characteristics of the bZIP members may form before the divergence of *Arabidopsis* and Cucurbitaceae since conserved domains were homologous in the same clade containing both *Arabidopsis* and Cucurbitaceae bZIP genes. However, it seemed that intraspecific duplication and parallel evolution of the bZIP family in each Cucurbitaceae has occurred afterward and led to the member variation in each clade. The majority of *bZIPs* in other clades originated from the whole genome duplication to evolve both generically and specifically in Cucurbitaceae. The results were in consistent with the conclusion in legumes (Wang et al., 2020).

In bottle gourd, 65 *LsbZIP* genes were undoubtably grouped into 12 subfamilies except clade M (**Table S2**, **Figure 4**). The general idea is that *bZIP* proteins in plant had extra functional conserved domains, in addition to specific *bZIP1* (PF00170) and *bZIP2* (PF07716) domains, participating in various biological processes (Jin et al., 2014). The domain basic leucine-zipper C terminal (bZIP_C) was identified only in clade C. Similar phenomena happened in clade G with G-box binding protein MFMR, and in clade D with seed dormancy control protein DOG1 as well (**Table S2**). The structure of motifs and exon-intron composition followed the classification pattern of phylogeny (**Figure 5**). Motif 2 and 6 were characteristic in clade D, composing DOG1 domain found in *Arabidopsis* (Dröge-Laser et al., 2018). The number of introns in *LsbZIPs* varied from 0 to 14, besides *LsbZIP8* owning incredible 21 introns (**Figure 5**). It was basically coincident with the results in *V. vinifera*, *Brachypodium distachyon*, *S. lycopersicum*, cassava and potato (Li et al., 2015a; Liu and Chu, 2015; Hu et al., 2016b; Mirzaei et al., 2020; Wang et al., 2020). Furthermore, most *bZIP* genes in clade F and S were prevalently intronless (**Figure 5**). It has been demonstrated that lack of introns contribute to accelerating the post-transcription in response to abiotic stresses (Zhou et al., 2018). And similar findings were observed in soybean and watermelon, revealing evolutionary imprint and functional divergence (Li et al., 2015b; Liu et al., 2017; Zhang et al., 2018; Yang et al., 2019). Compared to the intronless phenomenon, majority of clade D and G obviously possessed more introns (**Figure 5**). Previous researches suggested that the rate of evolution in genes without introns would increase after gene duplication (Lecharny et al., 2003; Lurin, 2004; Jain et al., 2006). It is deduced that *bZIPs* in clade D and G might derive earliest than other clades (Hu et al., 2016b). The orthologous genes in clade D and G shared overlaps in seven or eight species while genes in clade F shared overlaps in one or two species (**Figure 6A**, **Table S6**). It verified the speculation on the other side. Gene duplication is the main reason of genome evolution and expansion in multigene family (Si et al., 2019). In this study, 19 segmental duplication events in bottle gourd genome led to the expansion of bZIP family genes as in rice (Nijhawan et al., 2008), sorghum (Wang et al., 2011), maize (Wei et al., 2012), tomato (Li et al., 2015a) and peanut (Wang et al., 2019).

The genome-wide expression pattern of *LsbZIPs* exhibited tissue-specific trait in various organs in **Figure 8A**. For example, three genes including *LsbZIP17*, *LsbZIP43* and *LsbZIP63* were highly expressed and preferentially enriched in roots, indicating that they might play roles in root development. Similar tissue-specific pattern were obtained in many species such as apple (Li et al., 2016), grape (Liu et al., 2014), banana (Hu et al., 2016a), watermelon (Yang et al., 2019) and *Prunus mume* (Li et al., 2022). *LsbZIP50* and *LsbZIP24*, which were found to be highly expressed in all tissues except root, were orthologous of HY5 (At5g11260) and abscisic acid responsive elements-binding factor (ABF3, At4g34000), respectively (**Figure 7**; **Table S6**). The function of HY5 in numerous developmental processes has been well-established, like photomorphogenesis generation, chloroplast development and pigment accumulation (Gangappa and Botto, 2016; Yang et al., 2018). Likewise, *LsbZIPs* of clade A (52, 30), clade D (48, 61) and clade G (18, 5, 65) with high expression in all tissues were homologous to ABFs, TGA factors (TGAs) and G-box-binding factors (GBFs) in *Arabidopsis*. The ABFs have been found to act as the core of ABA signaling which counteract abiotic stresses (Banerjee and Roychoudhury, 2017). TGAs redundantly are regarded as vital transcriptional regulators by triggering systemic acquired resistance to interact with nonexpressor of pathogenesis-related genes 1 (NPR1), which is key gene in plant pathogen response (Fu and Dong, 2013). In addition, *LsbZIP37* showed cultivar-specific expression pattern in J104 and J129 which are relative resistant to cold stress, implying that *LsbZIP37* might participate in the regulation of cold stress response (**Figure 8B**).

Although bottle gourd is routinely used as a rootstock of other Cucurbitaceae crops due to its climate resiliency and high resistance to biotic stresses (Wang et al., 2020), the mechanism of which bottle gourd respond to cold stress has not been clearly understood. And plants have been found to evolve different strategies to adapt environmental stresses like cold (Xi et al; Wu et al., 2016; Xu et al., 2022). In the previous reports, a number of bZIPs including *OsbZIP73*, *OsbZIP52/RISBZ5*, *MdHY5* acts as positive or negative roles in response to cold stress (Liu et al., 2012; An et al., 2017; Liu et al., 2018). Based on the previous transcriptome profile, 19 *LsbZIP* genes were differentially expressed (12 up-regulated, 7 down-regulated) under cold stress (**Figure 9A**). Subsequently, 10 selected *LsbZIPs* were validated through RT-PCR at different time points after cold treatment (**Figure 9B**). *LsbZIP6* (clade K), *LsbZIP28* (clade B) and *LsbZIP61* (clade D) were quickly activated in response to cold stress. Their homologous genes in *Arabidopsis* contain three members of group B (*AtbZIP17*, *AtbZIP28*, *AtbZIP49*) and one member of group K (*AtbZIP60*), and have important effects on evolutionary conserved endoplasmic reticulum (ER) stress response (Howell, 2013). Transgenic plants overexpressed *AtbZIP17* showed salt tolerance and truncated *AtbZIP60* actively moved to the nucleus to regulate ER stress-genes (Iwata and Koizumi, 2005; Iwata et al., 2008; Liu et al., 2010). Therefore, B-bZIP *LsbZIP6* and K-bZIP *LsbZIP28* might converge on ER-stress pathways targeting specific gene sets like *AtbZIP60* and *AtbZIP28/bZIP17* were existed in heterodimer (Kim et al., 2018). *LsbZIP61* had orthologous relationship with TGA1, which interacts with NPR1 and induces apoplastic defense and ER stress response (Shearer et al., 2012; Wang and Fobert, 2013). G-bZIP *LsbZIP65*, which was orthologous to GBF1, exhibited a decline expression trend after cold treatment (**Figure 9B**). GBF1 is a well-known negative regulator of hypocotyl expansion (Gangappa et al., 2013), and can bind to the catalase2 promoter to inhibit the H_2_O_2_-scavenging activity, leading to ROS accumulation (Giri et al., 2017). The down-regulated expression of *LsbZIP65* might decrease the accumulation of H_2_O_2_ through less binding catalase2 promoter in order to endure cold stress in bottle gourd.

## Conclusion

In this study, we firstly identified and characterized 65 non-duplicated *bZIP* genes in *L. siceraria* and divided into 12 subfamilies at a genome-wide scale. The phylogenetic analysis based on 1235 *bZIP* sequences from 16 Cucurbitaceae revealed evolutionary convergence and divergence of *bZIP* gene family. The analysis of *LsbZIPs* domain, structure and orthologous relationships were consistent with the classification. The tissue-specific expression pattern of *LsbZIPs* indicated the pivotal roles in various physiological and biological processes. We elaborated the expression profiles of *LsbZIPs* in response to low-temperature stress, which provide valuable information of bZIPs under cold condition and strategies to breed cultivars conferred cold tolerance.

## Data availability statement

The datasets presented in this study can be found in online repositories. The names of the repository/repositories and accession number(s) can be found in the article/**Supplementary Material**.

## Author contributions

JW analyzed the data. YW and XinW conducted the qPCR analysis. JW drew the figures and edited the manuscript. BW, ZL and LZ contributed regents/materials/analysis tool. JW, GL and XiaW conceived and designed the study, obtained funds, and critically revised the manuscript. All authors contributed to the article and approved the submitted version.

## Supplementary material

Supplementary Figure 1 Phylogenetic analysis of bZIPs between two bottle gourd genomes (‘USVL1VR-Ls’ and ‘HZCG’).
